# Synthetic Calcite as a Scaffold for Osteoinductive Bone Substitutes

**DOI:** 10.1007/s10439-015-1520-3

**Published:** 2015-12-14

**Authors:** Anna Chróścicka, Zbigniew Jaegermann, Piotr Wychowański, Anna Ratajska, Jarosław Sadło, Grażyna Hoser, Sławomir Michałowski, Malgorzata Lewandowska-Szumiel

**Affiliations:** Department of Histology and Embryology, Center for Biostructure Research, Medical University of Warsaw, Chałubińskiego 5, 02-004 Warsaw, Poland; Centre for Preclinical Research and Technology, Banacha 1B, 02-097 Warsaw, Poland; Department of Biophysics and Human Physiology, Medical University of Warsaw, Chałubińskiego 5, 02-004 Warsaw, Poland; Department of Ceramic Technology, Institute of Ceramics and Building Materials, Postepu 9, 02-676 Warsaw, Poland; Department of Oral Surgery, Medical University of Warsaw, Nowogrodzka 59, 00-006 Warsaw, Poland; Department of Pathology, Center for Biostructure Research, Medical University of Warsaw, Chałubińskiego 5, 02-004 Warsaw, Poland; Institute of Nuclear Chemistry and Technology, Dorodna 16, 03-195 Warsaw, Poland; Laboratory of Flow Cytometry, Center of Postgraduate Medical Education, Marymoncka 99/103, 01-813 Warsaw, Poland

**Keywords:** Synthetic calcite, Bone tissue engineering, Human primary osteoblasts, Scaffold

## Abstract

Although a wide variety of biomaterials have been already proposed for use in bone tissue engineering, there is still need for man-made materials, which would combine support for osteogenesis with simplicity desirable for upscaling and costs reduction. In this study we have shown that synthetic calcite may serve as a scaffold for human osteoblasts transplantation. A simple dynamic system allows uniform and effective cell distribution. Cell viability and osteogenic phenotype were confirmed by XTT assay, alkaline phosphatase activity and selected osteoblast-specific genes expression. Extracellular matrix deposited by cells improved elasticity and made the whole system similar to the flexible composite material rather than to the brittle ceramic implants. It was revealed in the compression tests and also by the improved samples handling. Subcutaneous implantation of the cell-seeded calcite scaffolds to immunodeficient mice resulted in mineralized bone formation, which was confirmed histologically and by EPR analysis. The latter we propose as a method supplementary to histological analysis, for bone regeneration investigations. It specifically confirms the presence of bone mineral with a unique sensitivity and using bulk samples, which eliminates the risk of missing the material in the preparation. Our study resulted in development of a new osteogenic tissue engineered product based on man-made calcite.

## Introduction

Bone tissue engineering is one of the most intensively developing areas of regenerative medicine. That is because musculoskeletal tissues injuries are one of the most common traumas of both young and elderly patients due to the aging of the population combined with a rising popularity of the active lifestyle.^13^,^15^ Currently, there are many surgical procedures based on tissue transplantation as well as on the use of biomaterials available for the treatment of bone defects. Nevertheless, there are still huge expectations for new methods of bone supply and regeneration, free from the limitations of the currently used methods. A possibility to combine cells with the scaffolds made of biomaterials providing the biological activity of the implant, with no need to acquire a large amount of tissues for transplantation represents the unique advantage of tissue engineering. The two decades of research on tissue engineering have resulted in various types of biomaterials already proposed for use. However, they are still far from being faultless and the search for new inventions is actively ongoing.^21^ Current intensive research effort focuses on more advanced biomaterials intended to deliver drugs or growth factors, capable of responding to the local biochemical signals, like biosensors.^5^,^12^ On the other hand, complexity and high costs of the tissue engineered products (TEP) result in a gap between the research and clinics. Therefore, it is worth reflection on whether the easily available, relatively simple and as such—more affordable materials have been sufficiently exploited in tissue engineering. Among them, synthetic calcium carbonate might be taken into consideration as a suitable material for scaffold preparation, mainly for the purpose of bone tissue regeneration.

Encouraging outcomes of bone replacement using calcium carbonates have been already reported, but they refer only to the materials of natural origin.^1^,^4^,^8^ Both, results from the animal experiments and the examples from orthopedic and maxillofacial surgery in humans are available.^3^,^4^,^8^,^25^ Usually, the materials based on calcium carbonates are made of corals or mollusc shells.^4^,^16^,^22^ The main advantage of natural calcium carbonates is their resorbability.^4^,^8^ However, using natural materials has certain disadvantages, including the limited availability of their resources—some corals species are in danger of extinction^31^—and a very limited reproducibility of the materials composition and characteristics. Synthetic calcite scaffolds, as opposed to natural ones, could be well characterized, prepared with established and repetitive methods and would be widely available.

The core idea of this study is to propose synthetic—and as such, free from the limitations of natural materials—calcite scaffolds for bone tissue engineering. We aim to verify whether human osteogenic cells can be distributed within such scaffold, survive and express osteogenic activity. We also propose a new calcite-based TEP with osteogenic capacity for bone applications. The proof of concept *in vivo* observation of the calcite scaffolds enriched with human osteoblasts is performed using immunodeficient mice.

## Materials and Methods

### Cells used in Experiments

For development of calcite-based TEP, human bone derived cells (HBDCs) were used, based on the approval of the Bioethical Committee of the Medical University of Warsaw (Approval no: KB/74/2005; KB/116/2006).

HBDCs were isolated from the pieces of human bone removed from the bottom distal part of the long tight bone during knee joint alloplasty, which would otherwise be discarded. The donors, eight female patients, aged between 64 and 81, provided informed consent. Cells harvested from different donors were cultured separately, not pooled, and the population used in each particular experiment (first passage) always originated from one donor. HBDCs were isolated following the protocol for obtaining osteoblasts provided by Gallagher and colleagues.^7^ Briefly, soft tissue was removed from extracted bone pieces by scraping, and bone was cut into small pieces and treated with collagenase (Sigma, USA) overnight. Next, the bone pieces were put into the standard culture medium containing DMEM (Gibco, UK) enriched with 10% of heat-inactivated FCS (Gibco, UK), 1% antibiotic–antimycotic (Gibco, UK), 1% l-glutamine (Gibco, UK) and 100 *µ*M l-ascorbic acid 2-phosphate (Sigma, USA) and left in humidified air (5% CO_2_, 95% relative humidity) in order to allow osteoblasts to migrate from bone explants onto the surface of culture bottles (Nunc, Denmark). The medium was changed every 7 days. After confluence was reached, cells were detached by collagenase and trypsin (Gibco, UK) digestion, and used in the experiments. Culture medium used for experiments was enriched with HEPES (Gibco, UK).

For the experiments performed in order to optimize cell seeding and culture within the scaffolds -MG-63 (ATCC^®^ CRL-1427™) osteoblastic cell line was used. Cells from passage 101 were cultured in medium containing DMEM enriched with 10% of FCS, 1% antibiotic–antimycotic, 1% l-glutamine (without l-ascorbic acid 2-phosphate) in CO_2_ incubator. MG-63 osteoblasts were exposed to either dynamic or static conditions of cell culture within calcite scaffolds as described below.

### Scaffolds Description

Synthetic calcium carbonate scaffolds were manufactured so that the ultimate crystallographic form was calcite. The protocol was based on the previous studies, where various forms of calcium carbonates as well as the technical additives were tested.^19^ Porous calcite scaffolds were obtained from the material containing 99 wt% of calcium carbonate CaCO_3_ (POCh Gliwice) as a basic component and 1 wt% of lithium fluoride LiF (Sigma-Aldrich) as sintering activator. The material was prepared by dry mixing of calcium carbonate and lithium fluoride powders, followed by wet milling in rotary-vibration laboratory mill up to medium grain size *d*_50_ = 2.8 *µ*m. The material was dried in electric dryer, crumbled and pulverized by sieving.

Based on our previous investigations, we have used the method of mapping the matrix texture of polyurethane structural sponge in order to obtain porous material characterized by suitable and reproducible interconnected pore network (reticulate porous structure).^9^,^18^ Porous polymeric matrix of elastic polyurethane structural sponge of 45 ppi (pore per inch) density (Brösel) was used.

Samples in a shape of cylinder (15 mm in diameter and 5 mm in height) were prepared for *in vitro* experiments. For implantation, the cube samples were prepared (5 × 5 × 5 mm). All scaffolds were sintered in electric furnace in the temperature of 510 °C. Sintering was performed in air. Heating rate of 50 °C/h and 2 h dwell time in the temperature of 510 °C were applied to eliminate polyurethane sponge. As confirmed by thermal analysis, performed at the set-up stage, such firing conditions secured elimination of the foam without the need of any additional debinding treatment.

Physical properties of the obtained materials were characterized. The apparent density of the obtained materials, measured by geometrical method, ranged from 0.5 to 0.6 g/cm^3^. Total porosity of calcite material ranged from about 75–85% and compression strength from about 0.5 to 1.0 MPa. The size of the pores, was measured using microstructure images from stereoscopic and scanning microscopes and ranged from 300 to 350 *µ*m.^10^

In addition to the previously performed characterization of the material, qualitative analysis was carried out with the use of X-ray diffraction method in order to ensure the phase composition of the sintered samples prepared as a part of the TEP. Experimental setup included Bruker-AXS D8 DAVINCI diffractometer with Bragg-Brentano geometry. Measurement was conducted at a range of 5° to 120° 2*θ* (Cu Kα) with step size of 0.019° and measurement time of 1 s per step. Phase identification was carried out with the use of PDF-4+ 2014 ICDD database.

All scaffolds were sterilized by irradiation with electrons beam (10 MeV), at a dose of 25 kGy.

### Cell Seeding and Culture in the Scaffolds: Set-Up Observations Under Static vs. Dynamic Conditions

In order to establish protocol of effective cell seeding within the scaffolds, comparative observations of MG-63 cell line in static vs. dynamic culture were performed. For the first one, scaffolds were placed in the wells of a 24-well plate (Corning, USA) and seeded with MG-63 cells at a density of 10^6^ per scaffold. The cells were cultured in a standard culture medium (i.e. without l-ascorbic acid 2-phosphate) in a volume of 1.5 mL per scaffold. For a dynamic culture, commercially available Spinner Basket^®^ bioreactor (New Brunswick Scientific Company, USA) was used, as described elsewhere.^29^ Briefly, it is a closed system, where a continuous movement of culture medium is achieved by permanent mixing on a magnetic stirrer. The flow direction through the porous samples located between the two inner strainers inside the jar is enforced by the specific structure of the bioreactor. The dynamic observations were performed simultaneously to the static ones using the same cell population. After placing the scaffolds in a bioreactor, MG-63 osteoblasts suspended in 500 mL of a standard culture medium (9 × 10^5^ cells per sample) were added. The whole system was located in CO_2_ incubator. Dynamic and static cells culture conditions were maintained for 4 days. After this time the XTT test was performed, then the cells were fixed and stained with Hoechst 33258 (Sigma, USA) in order to localize cells nuclei.

### Preparing of TEP Based on Synthetic Calcite Scaffold and HBDCs in Dynamic Culture Condition

Prior to the experiment, the scaffolds were soaked and incubated in standard culture medium for 24 h. Next, samples were placed in bioreactor in a position, which ensured a permanent flow of the medium through the scaffolds. HBDCs were suspended in 0.5 L of culture medium (9 × 10^5^ cells per sample) and put into the bioreactor. The cells were cultured in CO_2_ incubator, where the whole system was placed. After 1 week the medium was changed for a fresh medium enriched with 10 nM dexamethazone (Sigma, USA), 10 nM vitamin D_3_ (Sigma, USA) and 10 mM beta-glycerophosphate (Sigma, USA). From this time point 50 mL of medium was changed twice a week. The samples in bioreactor were cultured for 5 weeks.

### *In vitro* Observations of HBDCs Activity and Phenotype in TEP

Cell viability on calcite scaffolds (*n* = 6) was examined on day 7, 14 and 35 using XTT assay (Sigma, USA) in each from the three independent experiments. This test is used in toxicology and is based on the ability of mitochondrial dehydrogenase enzymes in living cells to convert the XTT substrate [2,3-bis (2 methoxy-4-nitro-5-sulfophenyl)-5-[(phenyloamino) carboxyl]-2H-tetrazolium hydroxide] into a water-soluble formazan product. Although this test does not measure number of cells, its results are assumed to be proportional to the number of living cells.^23^ The results of the XTT test were presented as the absorbance level. The osteogenic capacity of the cells was confirmed by alkaline phosphatase activity test (ALP, Sigma USA) performed after 14 and 35 days of dynamic cell culture. This colorimetric assay is based on hydrolysis of *p*-nitrophenol phosphatase to *p*-nitrophenol.

ALP activity was normalized to cell number which was calculated on the basis of the XTT assay performed on the same cell population in parallel to the cell growth assay.

The phenotype of the HBDCs cultured on the surface of calcite scaffolds in the bioreactor was determined on the base of the osteogenic markers gene expression. The total mRNA was isolated from the HBDCs using RNeasy Micro Kit (Qiagen, Germany). The RT-PCR method (Titan One Tube RT-PCR Kit—Roche, Germany) was used to examine the expression of the genes for alkaline phosphatase (*ALP*), collagen type I (*Coll I*) and osteocalcin (*OC*). Their expression was evaluated in the material isolated from the samples maintained in the bioreactor for 14 and 35 days. The expression of each gene was measured independently on the material isolated from at least three scaffolds. mRNA, isolated from HBDCs cultured on tissue culture polystyrene served as a control. Additionally, the markers’ expression was evaluated in the material isolated from the sub-population of the HBDCs on the day of cell seeding (“0” control). As a housekeeping gene *GAPDH* (glyceraldehyde-3-phosphate dehydrogenase) was used. The gene-specific primers are shown in Table 1.

**Table 1:** Oligonucleotide primers sequence (5′–3′) used to RT-PCR reaction.

	Primers for PCR
GAPDH	5′-TCAAGGAAGCTACGGGCA- 5′-TGGCAGAAATTACACACACACAC-
ALP	5′-ATGTGGACTACCTATTGGGTCTC- 5′-GGGCCAGACCAAAGATAGAG-
Coll 1	5′-AGGGCTCCAACGAGATCGAGATCCG- 5′-TACAGGAAGCAGACAGGGCCAACGTCG-
OC	5′-TGAGAGCTCTCACACTCCTCGCCCTATTGG- 5′-GCTCCCAGCCATCGATACAGGTAGCGC-

HBDCs distribution within the calcite were examined using a fluorescent staining of cells nuclei (Hoechst) followed by a microscopic observation (NIKON ECLIPSE TE 2000-U) of the both, up and bottom surfaces of the samples as well as at the section in the middle of the scaffolds—after 7 and 35 days of a dynamic cell culture. Additionally, TEP obtained after 35 days long culture was observed in scanning electron microscope (HITACHI S-3500 N).

### Mechanical Strength of TEP

The mechanical properties of the developed scaffold-cells composites, were examined in a compression test using LR 10 K Material Testing Machine (Lloyd Instruments). The measuring head speed of 0.2 mm/min and the Nexygen 3.0 software were applied. In order to obtain a better distribution of pressure, cardboard spacers between the compression surfaces and the sample were applied. The test was carried out until the load value has decreased to 80% of the maximum load.

Cell–seeded scaffolds in shape of half of cylinder (15 mm in diameter and 5 mm in height) were tested after 5 weeks of a dynamic culture in bioreactor. Scaffolds placed in a 24-well plate and incubated in a culture medium without the addition of the cells served as a control. Mechanical tests were performed in the two independent experiments using the total number of the samples equal to 12.

### TEP Implantation to Animals

For *in vivo* observations, immunodeficient SCID mice were used because of the human-to-mouse xenogeneicity. The animals were exposed to cyclophosphamide, subcutaneously at 3 mg/animal, over two consecutive days before the experiment. The surgical procedure was conducted under general anesthesia. Nembutal (pentobarbital sodium) was administered intraperitoneally (20 *µ*g/g). Two symmetrical 5 mm skin dorsal cuts were performed 3 mm away from the mouse spine, starting just under the lower limb. Before subcutaneous scaffold implantation two pouches were made under the dorsal muscle layer by blunt dissection. One scaffold per pouch was inserted into each pocket.

Before implantation the 5/5/5 mm calcite samples were pre-cultured with HBDCs (3 × 10^5^ cells per scaffold) for 2 weeks in a Spinner Basket^®^ (C+ samples) or pre-wetted in a culture medium without cells (C− samples)—as a control. Implantation was always preceded by the verification of the cell viability by means of XTT assay. Each mouse was implanted with one C+ and one C− sample.

The experiment was performed twice. Each time, 22 C+ samples and 22 C− samples were implanted. Four and 12 weeks after implantation the animals were euthanized and both types of the implants (C+ and C−) with surrounding tissues were removed and proceeded for histological evaluation and EPR analysis. The animal experiments protocol was approved by the Animal Ethical Committee of the Medical University of Warsaw (Approval no: 29/29.11.2005).

### Histology and SEM Observation

The explants harvested after the observation period were fixed with 4% paraformaldehyde solution. Paraffin sections of 7 *µ*m were stained either with Hematoxylin & Eosin (HE) or with Sirius Red (SR)—for detection of the collagen orientation.

Scanning electron microscope observations of the explanted samples were performed. The outer and the inner surface of the TEP was observed.

### Electron Paramagnetic Resonance Analysis

Electron paramagnetic resonance (EPR) spectroscopy visualizes the EPR-active centers in the *γ*-irradiated samples. In this study it was applied to characterize EPR spectra with particular attention to the bone-characteristic EPR-signals in the explants harvested from animals. In order to generate active centers in the investigated samples, explants were exposed to the gamma irradiation using ^60^Co gamma source Issledovatiel with dose rate of 1 kGy/h and a total dose of 35 kGy. To distinguish the signals coming from the scaffolds and from the tissues, EPR spectra coming from the non-implanted scaffolds after radiation sterilization were measured as well.

EPR analysis was performed using a Bruker ESP300 X-band spectrometer equipped with high-sensitivity cavity ER 4108 TMH, Bruker ER035 M Gaussmeter and microwave frequency meter HP 5342A. For absolute g-value determination, a calibration using diphenylpicrylhydrazyl (DPPH) at 0.1 mW (*g* = 2.0036) was executed. The analysis was performed at the room temperature, with modulation frequency of 100 kHz, modulation amplitude of 0.1 mT and microwave power of 10 mW.

### Statistical Analysis

The data are expressed as mean ± standard deviation (SD). Statistical significance was assessed by analysis of variance (ANOVA) followed by Tukey *post hoc* test and Mann–Whitney test. Differences at *p* < 0.05 were considered to be statistically significant. The analysis was performed using GraphPad Prism software.

## Results

### Crystallographic form of Ceramic Part of the TEP - XRD Analysis

**Figure 1:**
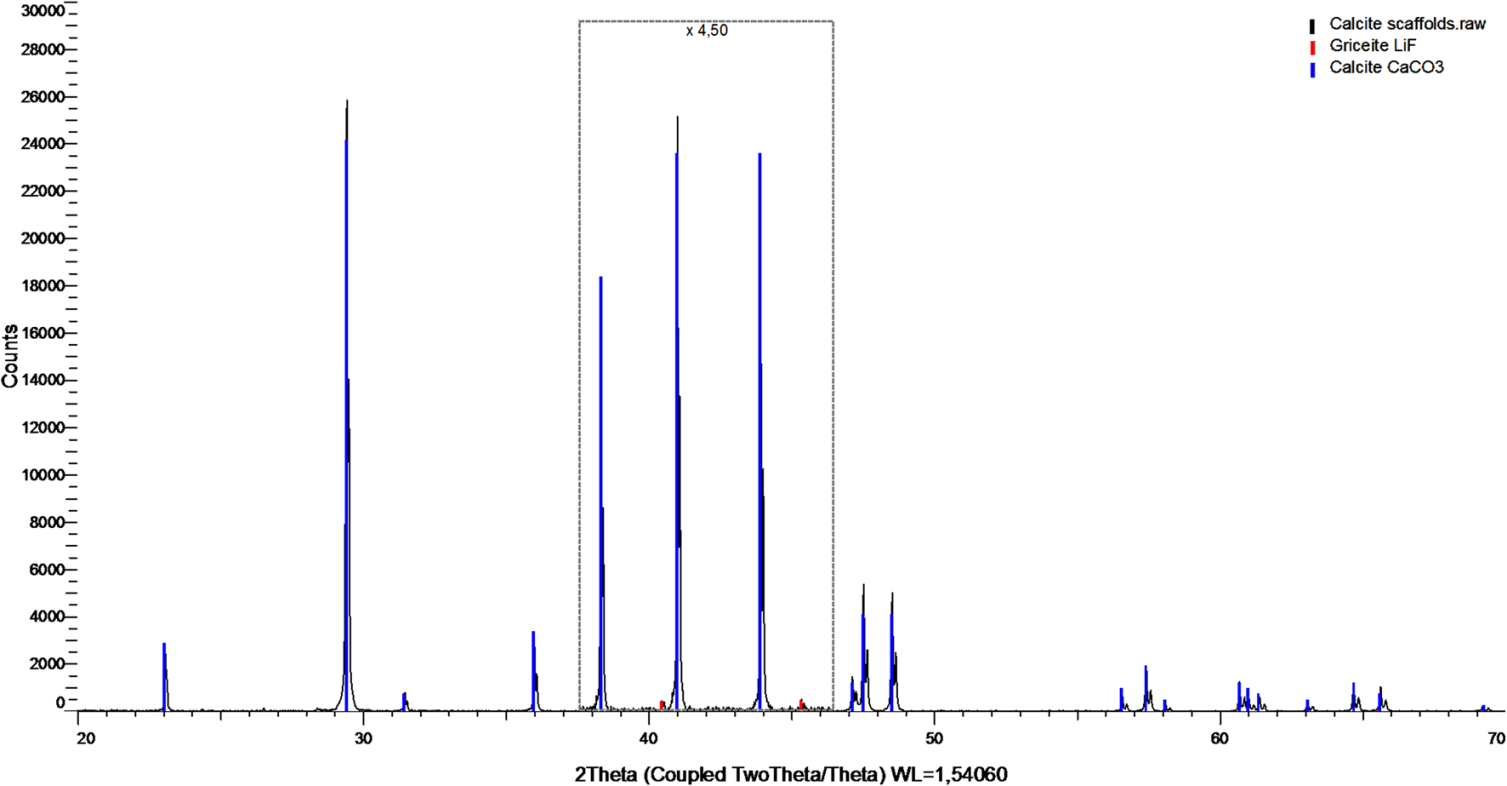
X-ray diffraction analysis of calcium carbonate scaffolds. Part of the graph has been increased ×4.5 to better illustrate the characteristic peaks of LiF. Identified phases: calcite CaCO_3_ (PDF 00-005-0586) and griceite LiF (PDF 00-004-0857)

In the XRD analysis, calcite was identified as the main phase and LiF as a trace in the investigated calcium carbonate scaffolds. There were no other crystallographic forms identified at the level of the sensitivity of the method, which is equal to 0.5% of the content. The results of the analysis are shown in Fig. 1.

### Observations in Cell Culture *In Vitro*

**Figure 2:**
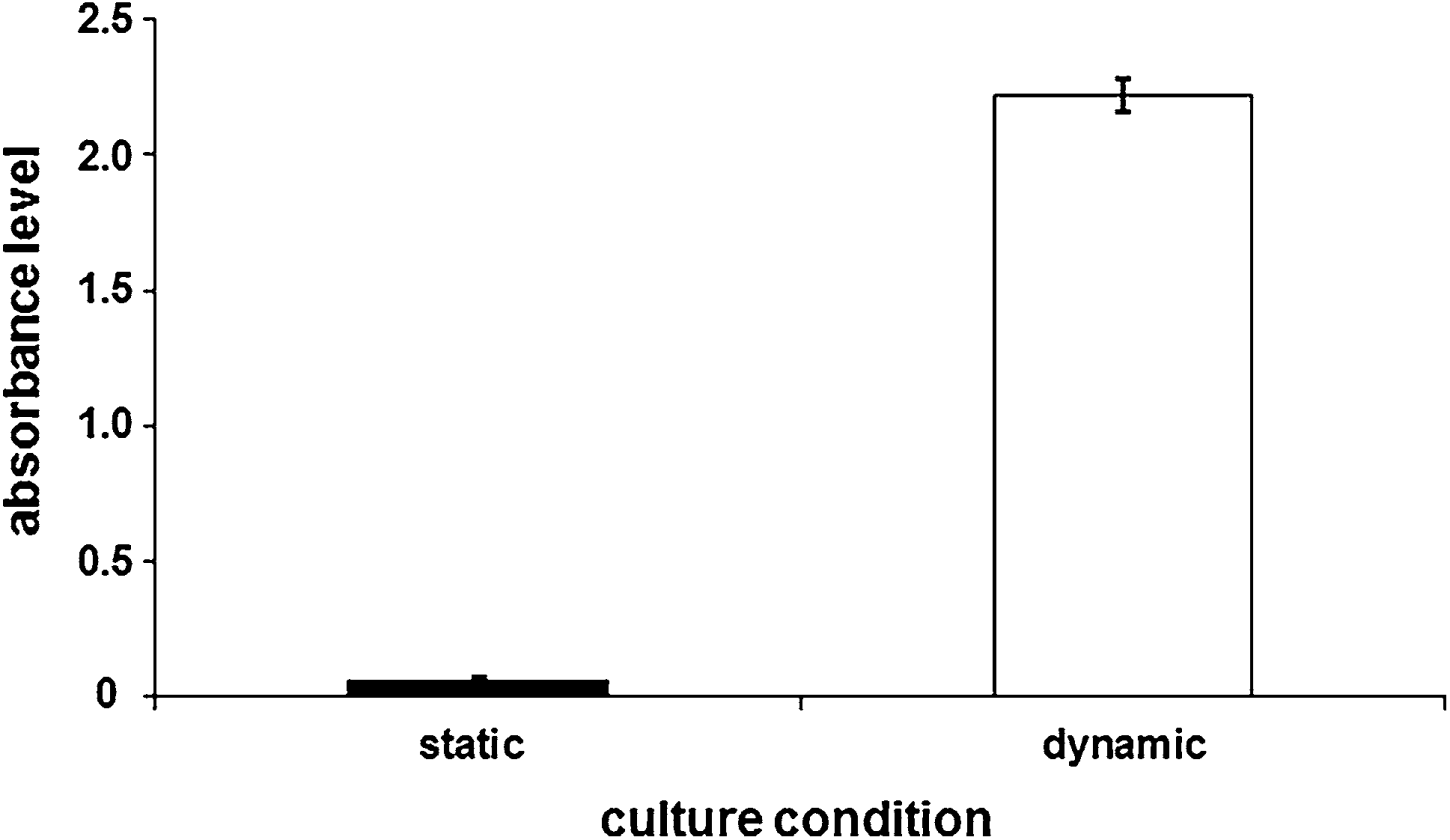
The results of the XTT test carried out after 4 days of static and dynamic cell culture. MG-63 cells were cultured on the surface of 3D calcite samples. Almost all cells cultured under static condition were dead. At the same time, there was a high number of viable cells cultured on the calcite scaffold under dynamic conditions in the bioreactor. The results are presented as the absorbance level: mean ± standard deviation (SD) (*n* = 6). The differences between the groups were statistically significant (*p* < 0.001) (Mann–Whitney test).

The influence of culture conditions on the viability of cells growing in contact with calcite scaffold was evaluated on the basis of XTT test. The test was performed after 4-day culture of MG-63 cells on the surface of calcite scaffolds. The obtained results indicate that almost all cells cultured under static condition in contact with the calcite samples, were dead. On the contrary, significantly higher (*p* < 0.001) cell viability on the scaffolds in the culture performed in the bioreactor was confirmed by means of the XTT assay, as shown in Fig. 2.

**Figure 3:**
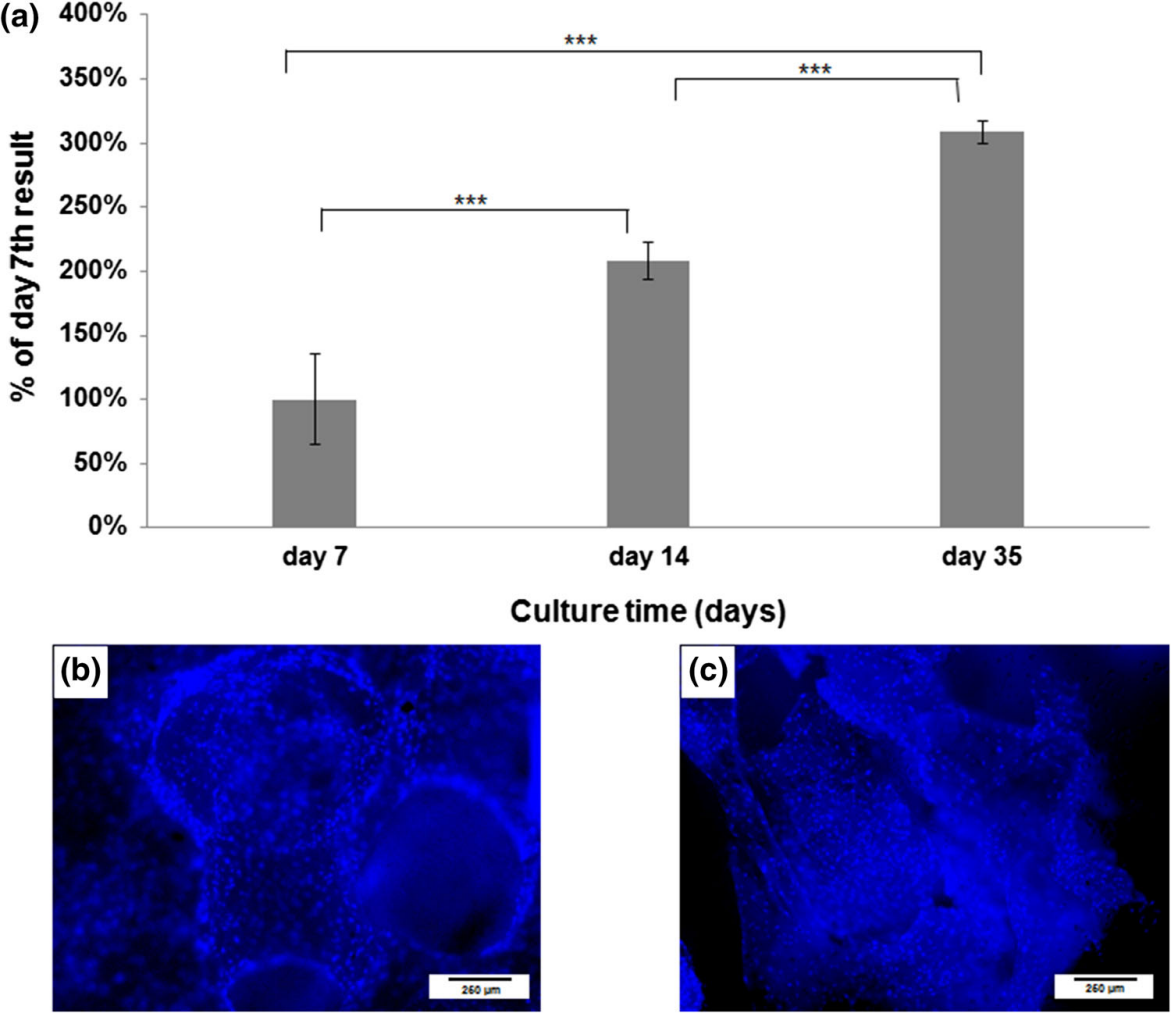
The results of the XTT test carried out on day 7, 14 and 35 of a dynamic HBDCs culture in the bioreactor (A)—the number of cells was significantly higher in each successive time point. The results are presented as a % of day 7th result, which was considered as 100%. Mean values ± standard deviation (SD) are shown (*n* = 6). Asterisks show significant differences (***p < 0.001) (Mann–Whitney test). Uniform cell distribution on the 3D calcite scaffold on day 7th (B—external surface of the scaffold) and on day 35th (C—interior of the scaffold) of dynamic culture. Cells were visualized after staining their nuclei with Hoechst.

Results obtained after 5 weeks of dynamic HBDCs culture within three dimensional calcite scaffolds indicated that cells were not only viable but also their number increased during the observation time (Fig. 3a). As revealed by XTT test, the population of living cells cultured on the calcite scaffolds by day 14 was significantly higher as compared to day 7, while on day 35 it was significantly higher as compared to the living cells populations both on day 7 and 14 (*p* < 0.001 in all cases). Moreover, the observations in fluorescent microscope revealed the uniform cell distribution within the calcite scaffolds—on the basis of the localization of cells nuclei visualized by a DNA-specific Hoechst staining on the outer and inner part of the scaffolds (Figs. 3b and 3c). Those observations were made after 7 and 35 days of cell culture.

**Figure 4:**
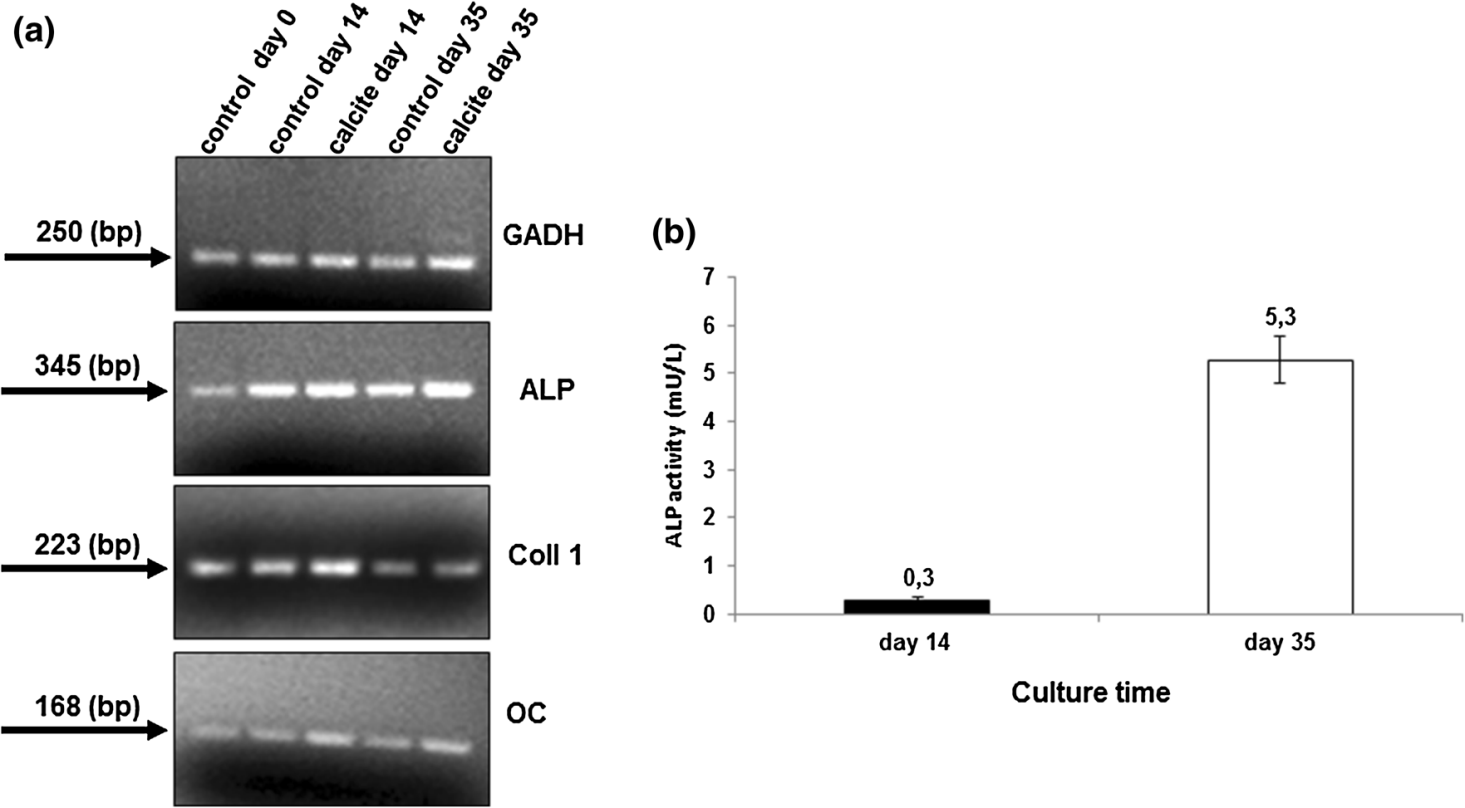
Representative samples of agarose gel electrophoresis profiles of RT-PCR products—GAPDH, ALP, COLL 1, OC specific genes primers were used. Analysis was performed on the selected time points in HBDCs cultured on the control surface and in the 3D calcite scaffolds. All products exhibited appropriate sizes visualized on ethidium bromide stained gels (a). ALP activity measured in cells cultured on 3D calcite scaffolds for 14 and 35 days. ALP activity has been shown in relation to a single cell in culture (b). Mean values ± standard deviation (SD) are shown (*n* = 6). The differences between the groups were statistically significant (*p* < 0.001) (Mann–Whitney test).

The ALP activity of cells cultured on the surface of calcite scaffolds was determined on day 14 and 35. The test results revealed almost 20-fold, statistically significant (*p* < 0.001) increase of ALP activity between day 14 and 35 of culture (Fig. 4b).

Additionally, the HBDCs phenotype was determined on the basis of the selected genes expression. The results of RT-PCR analysis are shown in Fig. 4a. Expression of the osteoblast-related genes: *ALP*, *Coll 1*, *OC* was confirmed in the both experimental time points, i.e. on day 14 and 35 of the HBDCs culture within the calcite scaffolds as well as in the control.

The overall characteristics of the samples at the end of the 5-week long observations was different for the scaffolds containing cells and those which were exposed to the culture medium for the same period of time but without cells. Tissue-like deposits were macroscopically visible within the pores of the C+ scaffolds.

**Figure 5:**
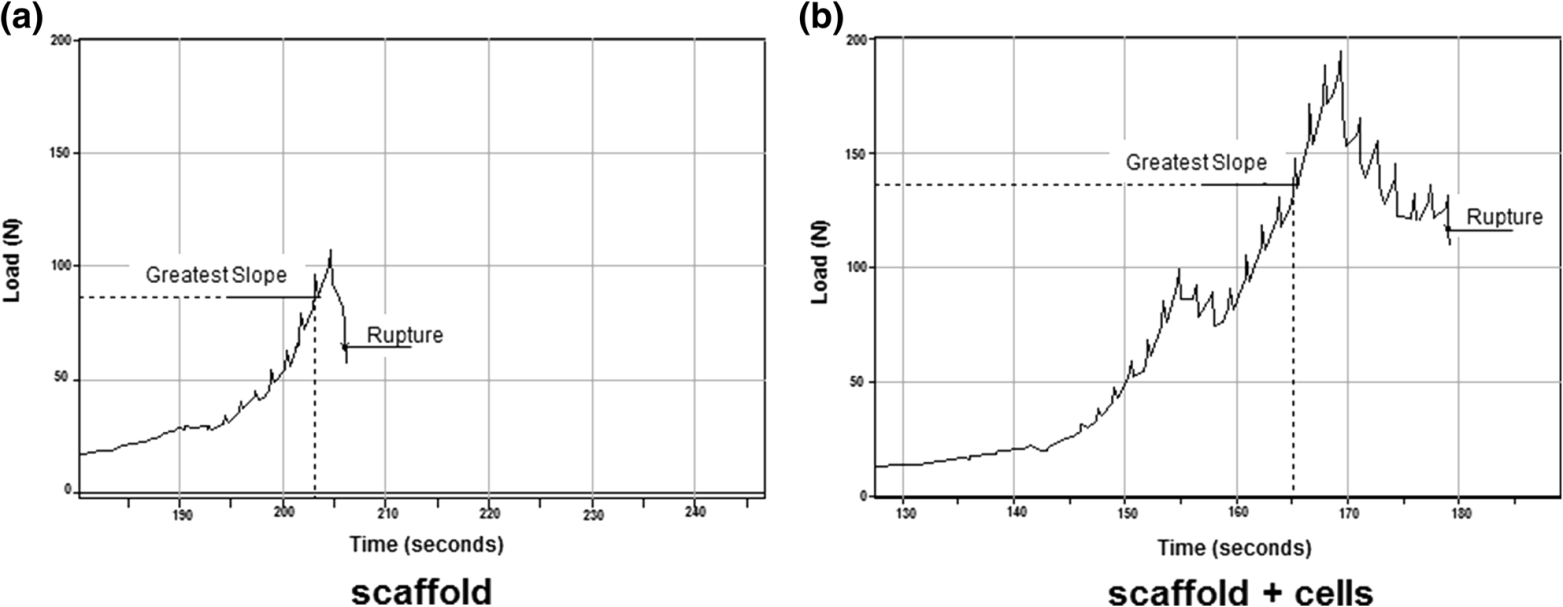
The graph shows the load vs. time curves from the mechanical assay. The test was conducted after 5 weeks long *in vitro* experiment on 3D calcite scaffolds without cells (a) and calcite scaffolds seeded with HBDCs (b). A characteristic feature of 3D calcite samples is charting a sharp decline in stress at the time of their destruction. The samples disintegrate completely (a). Scaffolds seeded with cells (b) behave differently. Characteristic property of these samples is that after reaching the maximum stress the decrease is gradual which is typical for composites and not for brittle materials.

**Figure 6:**
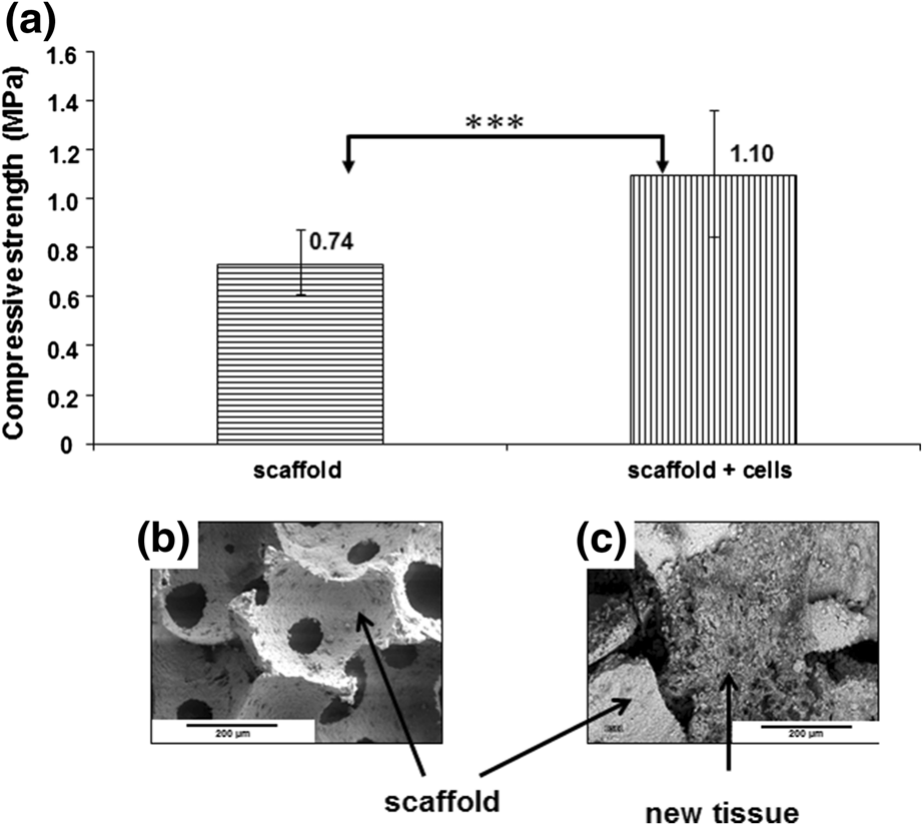
The graph shows the compressive strength of 3D calcite scaffolds and hybrid materials made up of calcite scaffold and HBDCs with extracellular matrix produced by the cells, measured after 35 days of *in vitro* culture. Mean values ± standard deviation (SD) are shown (*n* = 10). The differences between the groups were statistically significant (*p* < 0.001) (Mann–Whitney test) (a). SEM pictures show calcite scaffold structure (b) and new tissue produced by HBDCs during 35 days of dynamic culture on the 3D calcite scaffold (c).

At the end of the mechanical tests, i.e. at the moment when the load was dropped to the value of 80% of the maximum, the C− samples were completely disintegrated, while the C+ samples maintained cohesion, and they partially recovered their original form after removing from the machine. The load vs. time curves registered in the mechanical compression test revealed different profile of load resistance after reaching the maximum value (Fig. 5). For C+ samples the time needed to terminate the trial under the applied rules (load drop to 80%) was equal to 16 s in average, while for C− samples it took about 1 s. Such sharp decline in stress is characteristic for rigid porous materials. The compressive strength value determined in the compression test was equal to 1.1 MPa for the cells-containing scaffolds and equal to 0.75 MPa for the scaffolds without cells (Fig. 6a). This difference was statistically significant (*p* < 0.001). At the same time SEM observations made on the same samples revealed the presence of the extracellular matrix in the pores of the cells-containing scaffolds (Fig. 6c).

### *In Vivo* Observations

The general condition of the animals after surgery and during the whole observation period was good - there were no signs of infection or any other adverse reactions. Animals were healthy, skin cuts were healed and covered with fur. The calcite implants harvested after both 4 and 12 weeks were infiltrated by a vascularized tissue. There were no differences between C+ and C− samples under macroscopic observation. The new tissue was observed deep inside the pores of the scaffolds in both cases, as verified by SEM (data not shown).

**Figure 7:**
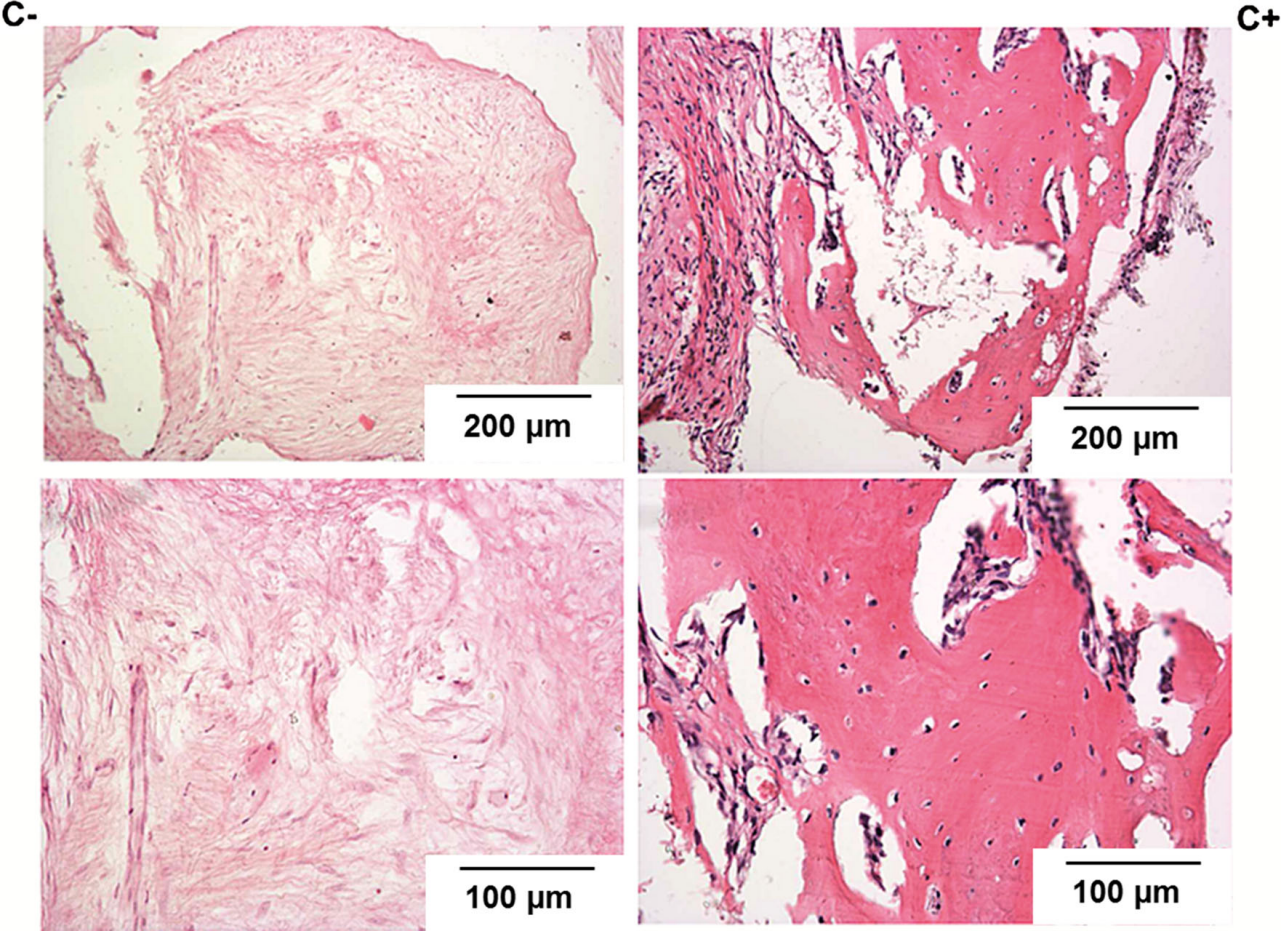
Histological pictures of the explants harvested 12 weeks after implantation (HE staining)—higher magnification at the bottom line. On the left side—pictures from the group where the naked, i.e. not-seeded with cells, scaffolds were implanted (C−). On the right side—pictures from the group where the implanted scaffolds were seeded with cells prior to implantation (C+). Only in calcites seeded with HBDCs before implantation bone tissue was found within the implants.

**Figure 8:**
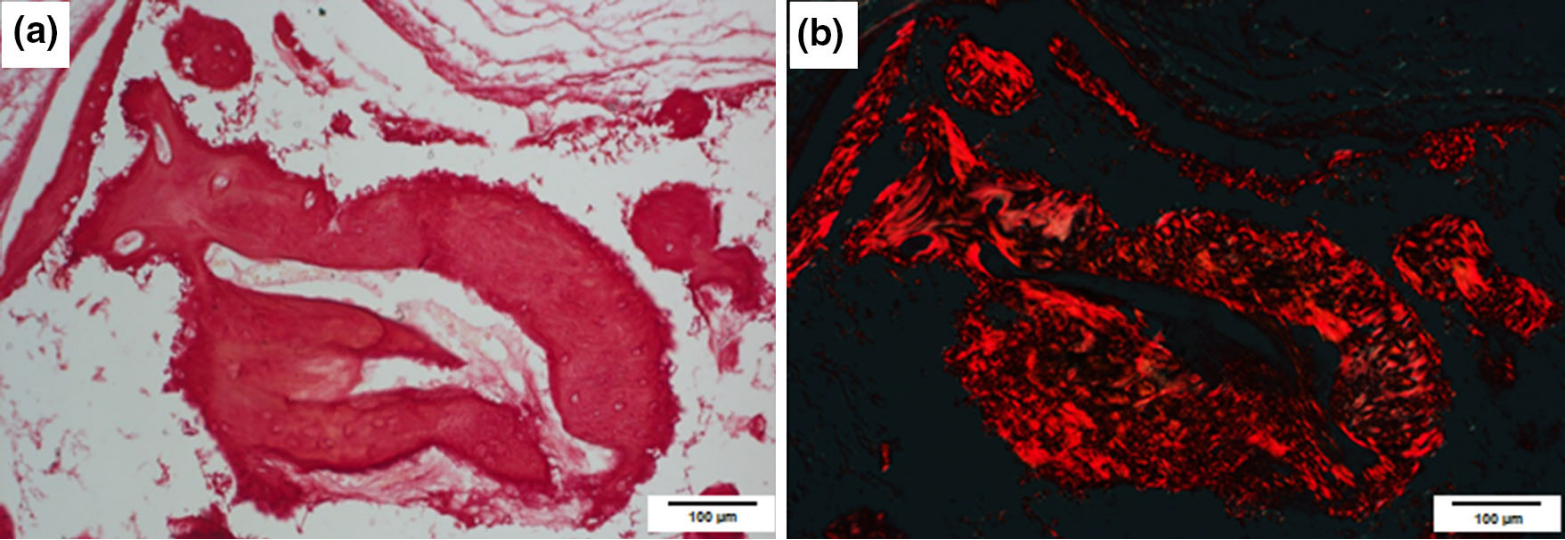
Histological pictures of a bone trabeculae, present in the explants harvested 12 weeks after implantation of the scaffolds which were seeded with cells prior to implantation. This bone trabeculae corresponded to the area of collagen deposition as has been confirmed by Sirius Red staining. Pictures A and B represent the same explant’s place stained with Sirius Red and visualized in transmitted light (a) and polarized light (b), respectively.

Histological observation performed on the samples harvested 4 and 12 weeks after implantation, revealed the presence of well-organized connective tissue inside the calcite scaffolds both in C+ and C− samples (H&E staining, Fig. 7). The fusiform (fibroblasts) cells and occasionally also the multinucleated giant cells were present. In focal areas of some samples slight inflammatory reaction was observed. This new connective tissue was rich in blood vessels in C+ and C− samples coming from both implantation times. Also collagen fibers were present in all implants with the exception of 4-week C− samples. After 12 weeks of implantation, in the scaffolds seeded with cells only, bone tissue was found within the implants (Fig. 7). Typical for bone trabeculae orientation of collagen fibers was visualized in polarized light on the sections stained by Sirius Red (Fig. 8).

**Figure 9:**
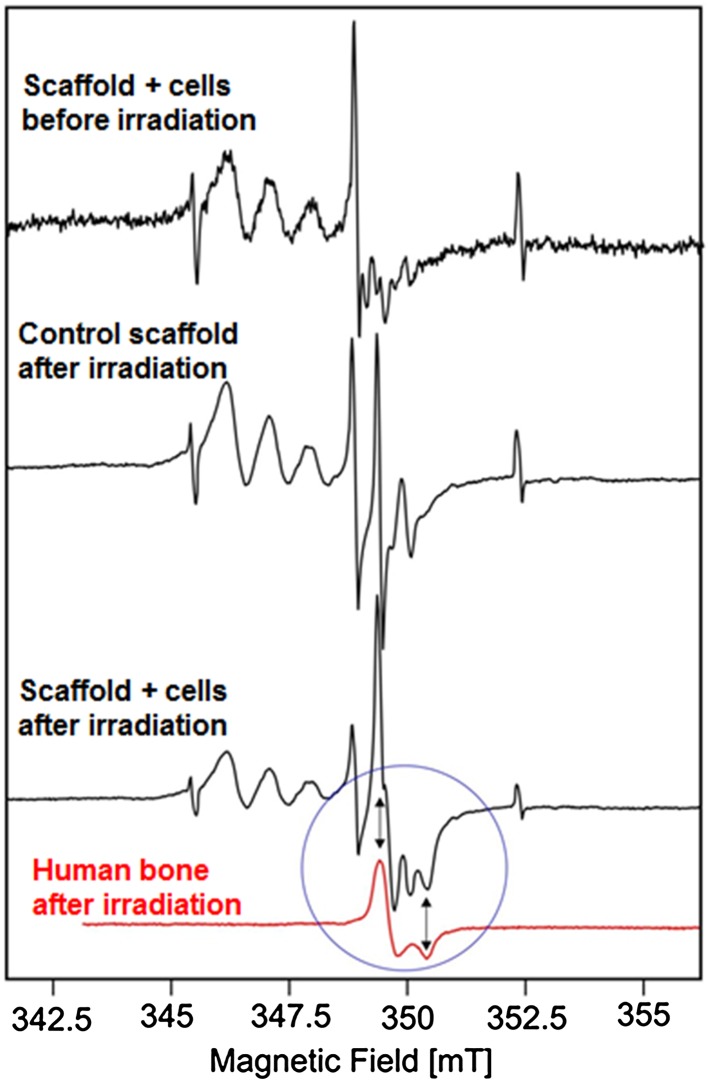
EPR spectra of calcite scaffolds. Arrows indicate perpendicular and parallel components of signal in bone (*g*(per) = 2.003, *g*(para) = 1.997).

EPR analysis was used to support the histological results and to confirm the presence of the mineralized bone tissue by detecting the signals specific for bone hydroxyapatite. After irradiation of the explants harvested from animal tissues, EPR spectrum contained all the signals coming from the scaffold, which were revealed by the analysis of the non-implanted irradiated scaffolds. However, in the C+ samples collected 12 weeks after implantation the additional singlet—characteristic for bone hydroxyapatite (*g*_*x*_ = 2.003, *g*_*y*_ = 1.997) was found (Fig. 9), while it was not detected either in the C− samples or in the samples harvested after 4 weeks.

## Discussion

Scaffolds designed for tissue engineering applications should not only fill in the missing tissue volume, but also serve as a substrate for cell delivery. Therefore, effective cells distribution and their good condition within the scaffold are crucial. Cytocompatibility of natural calcium carbonates was previously confirmed.^4^,^6^ Using synthetic calcite scaffolds for the purpose of bone tissue engineering is a new idea, thus, not much data on cell reaction in contact with such material is available. First data on synthetic calcium carbonate characteristics with regard to the cell response were published by F. Monchau et.al only in 2013.^20^ The authors have shown that proliferation and viability of mouse osteoblasts line MC3T3-E1 on the calcite substrate significantly diminished on day 3 and 6 as compared to the culture dish conditions but was comparable to those observed on the calcium-phosphate substrates. Reduced cell proliferation but higher ALP activity of MG-63 cell line was reported on a calcium carbonate coating on titanium surface when compared to uncovered titanium.^14^ The death of cells observed in our experiments in the static culture, may be related to elution of calcium ions from the residuals which may appear as a result of the intrasintering microdisintegration. Such residuals are difficult to remove even by prolonged washing—due to the high roughness and complexity of the material structure. Moreover, during sintering of a material consisting of calcium carbonate and lithium fluoride a slight decomposition of CaCO_3_ may occur under the influence of the temperature, as well as the formation of small amounts of amorphous Ca-rich phase. Both phenomena result in an increased release of calcium ions, which locally alkalizes the culture medium and reduces cell viability. The potential role of LiF is important for the further improvement of CaCO_3_-based TEPs and demands for further studies. Nevertheless, the problem of cytotoxicity induced with local alkalization of the medium was successfully resolved in our dynamic Spinner Basket^®^ system, which provided constant movement of the medium with a volume of 0.5 L. We obtained more than doubling and tripling of the population on day 14 and 35 in the culture on the calcite scaffold by applying this relatively simple system of dynamic culture (Figs. 2 and 3). Significant benefit of applying dynamic systems over the standard static culture condition is a known fact and was observed in many experiments before.^17^,^27^,^30^ It results also in the improved cells distribution in the 3D structures. In our experiments cells were observed not only on the upper and the bottom side of the scaffolds, but also on the entire surface of the cross-section of the samples (Figs. 3b and 3c). This is a result of effective cells nourishment due to the medium flow. It also confirms the suitable scaffold architecture with its open porosity and pore size of about 300–500 *µ*m—known to be appropriate for tissue ingrowth and bone regeneration after implantation *in vivo*.^2^ The results of the *in vivo* observations of the HBDCs -containing calcite-based system developed in our study seems encouraging. They revealed the formation of the mineralized bone tissue inside the pores of the scaffolds 12 weeks after subcutaneous implantation to immunodeficient SCID mice (Fig. 7). Since this effect was found only in the implants containing HBDCs seeded in the scaffolds, but not in the naked, i.e. non-seeded with cells scaffolds, it must have involved the osteogenic activity of the implanted cells. Their osteogenic capacity was confirmed *in vitro* prior to implantation. During 5-weeks long culture we observed significant increase of ALP activity in response to differentiation culture medium—which is widely accepted marker of the cells osteogenic potency.^7^,^11^ Moreover, the osteoblast-related genes expression, namely: *ALP, Collagen type I* and *Osteocalcin*—a specific marker of osteoblasts, was confirmed in HBDCs cultured within calcite scaffolds by means of RT-PCR (Fig. 4a). All those data confirm that HBDCs cultured on the synthetic calcite scaffolds under dynamic conditions save their osteogenic function.

Moreover, the developed scaffolds must have delivered the desirable ions to the implantation site, which allows to organize not only extracellular bone matrix of the appropriate protein content, but fully mineralized tissue. In our previous investigations, we confirmed osteogenic capacity of HBDCs implanted on the polyurethanes scaffolds in the analogous *in vivo* system, but we did not obtain mineralized bone tissue there.^29^ In the current study, when calcite scaffold is applied, the presence of the mineralized bone was proved both by histological evaluation (Figs. 7 and 8) and by the EPR study (Fig. 9). The latter we propose as a supplementary method, in parallel to the histological analysis, for the bone regeneration investigations—as the extremely credible bone mineral detection technique. EPR spectroscopy allows determination of the structure of radicals and EPR-active centers created in the irradiated samples. Its usefulness for bone tissue characterization comes from the fact, that in the biominerals containing hydroxyapatite, such as tooth enamel or bone, irradiated with ionizing radiation, characteristic paramagnetic centers are induced.^24^ They can be detected by EPR with a very high sensitivity, are extremely stable in time and their intensity responds in a controlled manner to the dosage of irradiation, which makes it useful for bone dating, dosimetry in tissues containing bone hydroxyapatites and detecting the remodeling of irradiated bone grafts in experimental systems.^26^,^28^

For characterization of bone TEPs, the two very important advantages of using EPR may be underlined. Firstly, it can be applied to the full volume sample, while being extremely sensitive at the same time. This helps to avoid artifacts that may arise from the omission of interesting pieces of tissues during sectioning, which can easily happen when the presence of the scaffold does not allow for serial sectioning. Although there is a broad range of methods to detect selected ions present in the sample, the EPR is superior to other techniques as it reveals the signal characteristics not for a group of ions, but just the bone hydroxyapatite. In our study EPR analysis confirmed the presence of the paramagnetic signal characteristic for bone mineral in the explants consisting of calcite scaffolds and HBDCs harvested 12 weeks after implantation. There was not such a signal neither in the samples which did not contain the human cells nor in the explants harvested earlier, i.e., 4 weeks after surgery, regardless of whether they contained HBDCs or not. These results are in an accordance with the histological evaluation and prove osteogenic potency of the calcite scaffolds enriched with human osteoblasts.

Subcutaneous osteogenesis of the cells-containing tissue engineering systems is not unique for the calcite-based systems. Similar results have been already published for calcium phosphate scaffolds,^32^ which are the most often studied biomaterials for bone reconstruction. Some studies show a promising results of the research performed on the calcium carbonates converted to hydroxyapatite by hydrothermal exchange.^31^ Our results prove the osteogenic potential of the product based on a simple synthetic calcite without any additional chemical treatment. Simplicity of the system may be crucial when transition from the bench to the clinical scale of the medical devices or medicinal products application is considered, and the manufacturing costs may limit the application. The technological regime for a single-phase material, such as calcite, may be less demanding as compared to the multiphase products, which seem to be the most required variant of calcium phosphates. While the technology of obtaining well-controlled ceramic-based scaffolds has long been mastered, a simplicity of the material may be a benefit, especially when up-scaling and reproducibility of the final product is considered.

Another issue important for the characteristics of the developed calcite-based system is its mechanical properties. The compressive strength of the naked, i.e. not seeded with cells scaffolds is low (Fig. 6)—as a consequence of high porosity of the samples. However, our results show that the prolonged—up to 35 days long culture of the cells distributed within the scaffold is possible. Both cell viability and osteogenic phenotype at this time point was confirmed (Figs. 3 and 4). Such prolonged culture results in obtaining a structure of a composite containing calcite and tissue-like structure—as visualized in SEM (Fig. 6). The mechanical tests confirmed a composite-like nature of the cells-enriched scaffolds. The compressive strength of such structure is slightly, but statistically significantly higher (*p* < 0.001) as compared to the naked scaffold. But what is even more important, characteristics of the destruction is different. In the case of brittle scaffolds, it is typical to observe a sharp decline in stress at the time of their destruction, as was demonstrated for scaffolds without cells. On the contrary, behavior of cell-enriched scaffolds in mechanical tests is characteristic for the polymer-ceramic composites, in which a porous brittle material is partially filled with the elastic one. Therefore, we postulate that the calcite-based scaffolds enriched with cells and supported by the cell-deposited ECM offer the improved handling of the ceramic-based bone grafts. They are much less brittle than ceramics alone and may be even slightly modified in shape in the operating theater, which is expected by surgeons. The osteogenic potency of such a system calls for further preclinical study. However, the osteogenic phenotype confirmed *in vitro* in the prolonged culture as well as the approved *in vivo* osteogenic potency of the analogous system after shorter culture seems highly encouraging.

## Conclusions

Synthetic calcite may be proposed as a material for scaffolds used in bone tissue engineering. We show that it maintains proliferative potency as well as osteogenic capacity of human osteoblasts distributed and grown within the scaffold in a dynamic culture. Osteogenic properties of the developed calcite-based TEP were confirmed *in vivo* in the subcutaneous model in immunodeficient mice as well. The applied scaffolds, which were shown to be overgrowth by a mineralized bone tissue after implantation to soft tissues, were not converted to hydroxyapatite by hydrothermal exchange, but were used after standard sintering. We underline the simplicity of this monophasic, easy to obtain substrate for cell transplantation, which maintain control and repeatable characteristics unlike the calcium carbonates of natural origin. We also developed the osteogenic bone substitute, supported by the extracellular matrix deposited by the cells in the prolonged culture. It has a composite-like mechanical characteristics instead of a brittle one as well as the improved surgical handling.

We postulate applying EPR analysis, which may confirm the presence of mineralized bone with a unique sensitivity and free from the risk of missing the material due to the preparation, as a method supplementary to the histological analysis, for bone regeneration investigations.
